# Air column in esophagus and symptoms of gastroesophageal reflux disease

**DOI:** 10.1186/1471-2342-12-2

**Published:** 2012-01-25

**Authors:** Alijavad Moosavi, Hanieh Raji, Mojtaba Teimoori, Shadi Ghourchian

**Affiliations:** 1 Department of pulmonary diseases, Rasoul-e-Akram Hospital, Tehran University of Medical Sciences, Sattakhan street, Tehran(1445613131), Iran; 2 Department of pulmonary diseases, Jundishopur University of Medical Sciences, Golestan boulevard, Ahvaz, Iran; 3 Nephrology and Urology Research Center, Medical Faculty, Baqyatallah University of Medical Sciences, Mollasadra street, Tehran, Iran; 4 Medical student, Scientific students' research center of Tehran University of Medical Sciences, DDRI of Rasoul hospital, Sattakhan street, Tehran (1445613131), Iran

**Keywords:** Gastroesophageal Reflux Disease, chest computed tomography, radiographic anatomy

## Abstract

**Background:**

During imaging of the normal esophagus, air is often detected. The purpose of this study was to determine the correlation between the appearance of air bubbles on imaging and Gastroesophageal Reflux Disease (GERD) symptoms.

**Methods:**

The cross-sectional imaging study was conducted at Rasole Akram Hospital, Tehran, Iran. A total of 44 patients underwent X-ray computed tomography (CT) scanning; the presence of air in the esophagus and visible on CT imaging was scrutinized.

**Results:**

The average age of the subjects was 59 and the male to female ratio was 0.83. We found a significant relationship between the presence of GERD symptoms, the size of air bubbles and esophageal dilation (ED) on the CT scan.

**Conclusions:**

Air bubbles in the esophagus may be seen frequently in CT scans, but their size and location can vary. The GERD symptoms can arise when a small diameter air column is present within the esophagus, especially in the middle and lower parts.

## Background

Gastroesophageal Reflux Disease (GERD) is disruptive and places a great clinical and economic burden on patients and society as a whole [1]. A recent study estimated that 20% of the adult US population experience GERD-related symptoms at least once a week [2]. The disease is sometimes accompanied by extraluminal symptoms such as chronic cough, laryngitis, asthma and sinusitis [3]. GERD is one of the most common diseases that can be treated in many of those patients who suffer from it [4].

Air is usually seen in radiological exams of the normal esophagus, but the extent and distribution of air has not been well described, and there is a paucity of data [5]. Previously Proto showed air seen in 36% of normal chest radiograph and then in a study [6] reported that an air column is visible in 64% of CT scans of the normal esophagus. Bhalla and Silver [7] defined esophageal dilatation (ED) as an air column greater than 10 mm in the coronal plane. Ponce revealed that increase in esophageal diameter in is associated with greater disease evolution [8]. Halber have showed that air in the esophagus is a normal finding [9]. However, data to confirm these findings are scarce.

Therefore, the aim of this study is to identify and explore any relationships between GERD symptoms in the patients' history and the presence of air bubbles in the esophagus. We assessed the correlation between the size, number and position of any air bubbles, and the presence of GERD symptoms.

## Methods

### Study population

Our cross-sectional imaging study was conducted at Rasole Akram Hospital of Tehran University of Medical Sciences (TUMS), Tehran, Iran. The study was carried out in December of 2009 to May of 2010. A total of 44 patients underwent CT scanning as part of the clinical care of their pulmonary disease. Approval for the research was confirmed by the TUMS Ethics Committee.

### Study design

The study patients were selected from a group who were referred to the lung ward of our hospital. Since all of the cases were referred, it was expected that some of the patients' symptoms were caused by their main condition and so those patients were excluded. One exception was coughing, which was so prevalent that it could not be considered as an exclusion criterion in all patients; other investigated symptoms were all characteristic of upper gastrointestinal involvement. In addition, patients with a cough that was essentially related to lung disease were excluded as much as possible. Subsequently, the incidence of coughing related to respiratory disease was 30%. Because of the low prevalence of some symptoms in our study sample, e.g. nausea, some characteristics were omitted before performing the analysis.

### Data collection

We designed a check list to include all major and minor symptoms of GERD, such as epigastric symptoms; chest pain; difficulty swallowing; chronic laryngitis and/or sore throat; a chronic cough, regurgitation, heartburn, toothache following the consumption of cold or hot food, frequent pulmonary problems, post-nasal discharge (PND), chronic sinusitis, frequent and resistant nausea, gastrointestinal bleeding, history of pregnancy, smoking or of taking *proton pump inhibitors (PPIs) *for a long time, a history of hiatal hernia (HH) or of any other diseases.

### Definition

The CT images were reviewed by one of the authors, who is a trained pulmonologist in comparing the CT scans by unique criteria. The purpose of choosing different sizes and different numbers of axial images with air bubbles was to determine the presence or absence of bubbles in different parts of the esophagus with GERD symptoms. In addition, patients who had air columns in all axial images of CT were noted. The presence of air was characterized by its diameter, quantity, its location in the CT scan, and the presence or absence of ED. Because ED is not a recognized condition by standard clinical texts, we compared our findings with the criteria for a normal air column in the esophagus as determined by Dean [10]

### Radiologic measurement

To match the sizes of air columns with the sizes determined in 2009, we used a c-100 scanner (cine-CT; C-100 Scanner, Imatron, San Francisco, CA), using 1 to 2 mm collimation. Images were reconstructed to a 512-pixel matrix using a sharp kernel and a 26 cm display field of view. The standard mediastinal window (width, 396 HU; level, 44 HU) and lung window (width, 1465 HU; level, -498 HU) were used. Following the guidelines given by Schraufnagel [10], the assessments were all limited to the thoracic esophagus. Because the normal air column was measured in separate parts of the esophagus by CT, we divided the esophagus into 3 sections including the supra ventricle (SV), ventricle (CV) and ventricle to lower esophageal sphincter (V-LES). ED was defined as the presence of air bubbles greater than 10 mm in the SV and CV. Furthermore, air bubbles > 15 mm were named ED in V-LES. [11], we considered presence of ED: yes/no question for a diameter greater than 15 mm, and their size: the exact quantitative size not yes/no question for ED of air bubbles.

### Statistical analysis

Our analysis was done using SPSS software, version 17. We used frequency tests to quantify the frequency of men and women in our study population, and the frequency of symptoms among each gender.

To understand if there is any relationship between gender and the existence of ED in different segments of the esophagus, we used the chi-square test or Fisher's exact test when needed. Other qualitative variables were evaluated by a chi-square test as well, including epigastric symptoms such as chest pain, a chronic cough, regurgitation, heartburn, frequent pulmonary problems, frequent and resistant nausea, and a history of pregnancy, smoking, or a history of taking *PPIs *for two last weeks before imaging. Among all characteristics, the imminent presence of notable ED in V-LES was found in patients previously identified by means of endoscopy as suffering from HH. The expressed qualitative variables were also counted by a t-test, so that we could determine the correlation between symptoms and the sizes and number of bubbles in each segment, and in the whole esophagus. Some of these 9 variables had non-parametrical distributions, and were analyzed by the Mann-Whitney test. A P- Value > 0.05 was considered to be significant, and confidence interval (CI) was 95%.

## Results

### Demographic Data

Forty-four patients who had undergone a CT scan for their respiratory disease were evaluated. The average age of the patients was 59.31 ± 15.98 years and the male to female ratio was 0.83 (37 men and 8 women). Out of 44 patients, 11 (25%) had been taking omeprazole, and of these, 6 patients (54%) were female. In addition, of these patients, 5 (11.4%) were known by means of endoscopy to be HH cases, and 2 patients (40%) were female. Statistics showed that of the 20 patients (45.5%) with heartburn, 9 patients (45%) were female. Chronic cough was positive in 29 patients (65.9%), and of these, 11 patients (37.9%) had a history of respiratory or other related diseases.

### Air bubbles

**Figure 1:**
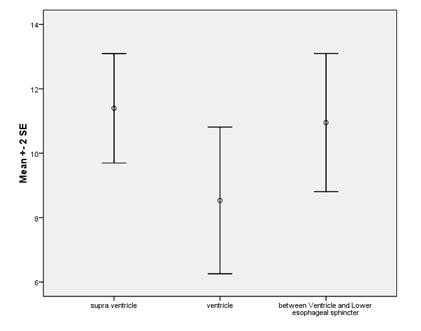
**The correlation between sizes (mm) and location of the esophageal blobs**.

**Figure 2:**
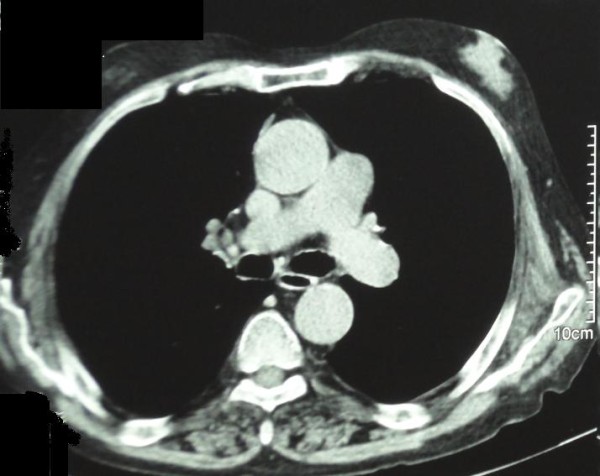
**CT scan illustrate size taken at full length of esophagus in 75-year-old man**. Greatest direct distance of air bubble of esophagus was measured. Recording line does not included soft tissue. The greatest space between its walls was measured. Scale is in millimeters.

**Figure 3:**
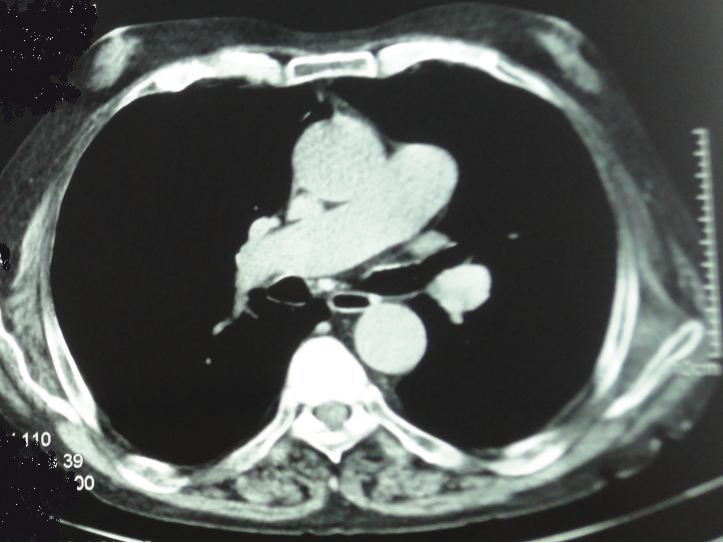
**Lumina of air bubble going down thorax in previous patient as in Figure 1**. This series of CT scans shows landmarks for distance down esophagus.

The mean size of the bubbles was 11.44 ± 5.51 mm in SV, 8.53 ± 7.47 mm in VC, 10.70 ± 7.13 mm in V-LES and 30.88 ± 14.95 mm in the whole of the esophagus. (Figure 1, 2 and 3)

Of all the cases, 7 patients (16.3%) had ED in the whole esophagus. Of them, 71.4% (5 patients) were women. In other patients with sporadic involvement, however, the frequency of men and women was the same (p > 0.05).

There were no correlation test between the sizes of the bubbles in SV and CV (p = 0.13) but we see the significant correlation between SV and V-LES (p = 0.006), and between CV and V-LES (p = 0.033).

### Correlations of Air bubbles Upper Gastrointestinal symptoms

After analyzing all symptoms and relative characteristics, we found a significant relationship between the presence of ED in V-LES and heartburn (32.3% versus 76.9% in patients with and without ED, p = 0.007, Odds Ratio [OR] = 7). Heartburn in patients whose CT included an air column in all axial images was significantly more prevalent than in the group whose CT revealed just some sporadic bubbles (P = 0.038 and OR = 9.429), (Tables 1, 2, 3).

**Table 1:** Correlation between sizes of blobs and frequencies of characteristics

characteristics	SV	CV	V-LES	Total**
**Chronic coughs**	P = 0.119 *(mean of positives = 12.37 ± 5.52, mean of negatives = 9.63 ± 5.21)	P = 0.422 (mean of positives = 9.21 ± 7.40, mean of negatives = 7.26 ± 7.69)	P = 0.029 (mean of positives = 12.37 ± 6.69, mean of negatives = 7.46 ± 7.05)	P = 0.076 (mean of positives = 34.37 ± 14.37, mean of negatives = 24.36 ± 14.21)

**Smoking**	P = 0.94 (mean of positives = 13.30 ± 4.80, mean of negatives = 10.32 ± 5.72)	P = 0.780 (mean of positives = 8.30 ± 8.17, mean of negatives = 8.98 ± 7.15)	P = 0.151 (mean of positives = 13.06 ± 6.94, mean of negatives = 9.82 ± 6.92)	P = 0.253 (mean of positives = 34.66 ± 17.21, mean of negatives = 29.37 ± 13.45)

**History of omeprazole**	P = 0.214 (mean of positives = 9.63 ± 5.23, mean of negatives = 12.04 ± 5.55)	P = 0.493 (mean of positives = 7.181 ± 7.53, mean of negatives = 9.00 ± 7.51)	P = 0.710 (mean of positives = 10.00 ± 6.03, mean of negatives = 10.93 ± 7.54)	P = 0.537 (mean of positives = 26.81 ± 12.31, mean of negatives = 32.28 ± 15.68)

**Hiatal hernia**	P = 0.207 (mean of positives = 14.40 ± 4.33, mean of negatives = 11.06 ± 5.58)	P = 0.172 (mean of positives = 4.40 ± 6.06, mean of negatives = 9.07 ± 7.53)	P < 0.001(considered as a huge ED) (mean of positives = 20 ± 0.00, mean of negatives = 9.51 ± 6.69)	P = 0.869 (mean of positives = 38.80 ± 7.69, mean of negatives = 29.84 ± 15.41)

**Heartburn**	P = 0.040 (mean of positives = 13.30 ± 5.91, mean of negatives = 9.89 ± 4.75)	P = 0.920 (mean of positives = 8.5 ± 8.98, mean of negatives = 8.56 ± 6.07)	P = 0.010 (mean of positives = 13.67 ± 6.71, mean of negatives = 8.22 ± 6.62)	P = 0.230 (mean of positives = 35.47 ± 17.22, mean of negatives = 26.89 ± 11.60)

**Epigastric pain**	P = 0.665 (mean of positives = 11.97 ± 7.39, mean of negatives = 11.11 ± 4.05)	P = 0.905 (mean of positives = 8.80 ± 8.82, mean of negatives = 8.42 ± 6.62)	P = 0.023 (mean of positives = 12.97 ± 6.95, mean of negatives = 9.27 ± 7)	P = 0.688 (mean of positives = 33.64 ± 18.23, mean of negatives = 29.07 ± 12.41)

**Regurgitation**	P = 0.417 (mean of positives = 12.04 ± 5.76, mean of negatives = 10.65 ± 5.22)	P = 0.276 (mean of positives = 9.6 ± 7.90, mean of negatives = 7.05 ± 6.76)	P = 0.007 (mean of positives = 13.18 ± 6.12, mean of negatives = 7.44 ± 7.21)	P = 0.216 (mean of positives = 34.82 ± 15.78, mean of negatives = 25.41 ± 12.08)

*mean of positives shows the mean of blob size in the cases that had the characteristic and mean of negatives shows the mean of blob size in the cases that didn't have it.

**the summation of sizes of blobs in whole of esophagus.

**Table 2:** Frequencies and comparison between symptoms and relevant characteristics with the presence of esophageal dilation.

characteristics	presence of esophageal dilation comparing with the reference values/yes, no question					
	
	SV		CV		V-LES	
	
	ED-** 20.50%	ED+ 79.50%	ED- 41.90%	ED+ 70.50%	ED- 29.50%	ED+
Chronic coughs						

% *	44.4%	71.4%	55.6%	72%	58.1%	84.6%

P-value	0.235		0.264		0.162	

Smoking						

%	11.1%	41.2%	41.2%	32%	30%	46.2%

P-value	0.129		0.542		0.324	

History of taking proton						

pump inhibitors						

%	22.2%	25.7%	33.3%	20%	25.8%	23.1%

P-value	1		0.48		1	

Hiatal hernia						

%	0%	14.3%	16.7%	8%	0%	38.5%

P-value	0.566		0.634		0.01	

Heartburn						

%	22.1%	51.4%	61.1%	36%	32.3%	76.9%

P-value	0.15		0.103		0.007	

Epigastric pain						

%	33.3%	40%	44.4%	36%	35.5%	46.2%

P-value	1		0.576		0.507	

Regurgitation						

%	44.4%	60%	55.6%	60%	48.4%	76.9%

P-value	0.467		0.771		0.081	

History of pregnancy(in women)						

%	80%	63.2%	83.3%	58.8%	70.6%	57.1%

P-value	0.702		0.407		0.738	

* Percent of patients who presented symptom (positive symptom).

**- shows cases with no ED and + shows cases wi

**Table 3:** Frequencies of each characteristic in cases with sporadic air blobs and cases with air column in whole esophagus

characteristics		Chronic coughs %**	Smoking %	History of omeprazole %	Hiatal hernia %	Heartburn %	Epigastric pain %	Regurgitation %	History of pregnancy (in women) %
The presence of air column whole the esophagus (not necessarily ED in whole)	-*	61.1%	34.3%	25%	13.9%	38.9%	36.1%	52.8%	72.2%
	
	+	85.7%	42.9%	28.6%	0%	85.7%	57.1%	85.7%	40%
	
	P-value	0.391	0.686	1	0.572	0.038	0.407	0.209	1

*- shows cases with sporadic air blobs and + shows cases with air column in whole esophagus.

** Percent of patients who presented symptom (positive symptom).

We found a significant relationship between main upper gastrointestinal symptoms accounting mainly for GERD and the sizes of bubbles, to the extent that they caused ED. Aside from the significant values of the qualitative characteristics, the relationship between heartburn and the appearance of ED > 15 mm in V-LES and also the presence of ED in all segments of the esophagus were highly significant. (Table 2, Table 3), Furthermore, a significant correlation was detected between the size of the air pockets in V-LES and heartburn (p = 0.010) (Table 1). The specific correlation between the two expressed characteristics was manifested by the prominent positive mean. The mean size of bubbles in patients who had heartburn was 13.67 ± 6.71 mm, and in patients who had no heartburn, it was 8.22 ± 6.62 mm. This confirmed the correlation between heartburn and an increase in the sizes of air pockets in V-LES (p = 0.010).

Although the P-value of the correlation between regurgitation and the presence of ED in V-LES was not statistically significant (48.4% versus 76.9% in patient with and without ED, p = 0.081), there was a significant correlation between the sizes of the bubbles in V-LES and the presence of regurgitation (13.18 ± 6.12 mm versus 7.44 ± 7.21 mm air bubble size in patients with and without regurgitation, p = 0.007). Because of the predominantly positive findings of the presence of regurgitation, the correlation between the sizes of the bubbles and regurgitation was confirmed.

There was no significant correlation between regurgitation and the sizes of the bubbles in other parts of the esophagus, including CV and SV (12.04 ± 5.76 mm versus 9.60 ± 7.90 mm in SV and CV, p = 0.41 and p = 0.27 in SV and CV respectively).

## Discussions and Conclusions

In a CT scan, air bubbles are frequently seen, and their sizes and locations may vary [10]. In this study, we found there was a relationship between the presence of air bubbles and some GERD symptoms such as heartburn.

As heartburn is considered to be the most prevalent problem in patients with recognized GERD, an increase in the probability of heartburn due to an increase in the diameter of air columns in most parts of the esophagus motivates widely different discussions. The dilation of the esophagus in V-LES can be ascribed to the probable concomitancy of lower esophageal sphincter (LES) dysfunction that can cause GERD symptoms. However, the presence of an air column in the whole esophagus and also the significant correlation between the sizes of bubbles and heartburn cannot be justified fully by our present knowledge, especially in SV. Furthermore in our study, there was no history of scleroderma or other diseases which can dilate the esophagus. While the average age of our patients was 59, ED could not be attributed to aging processes. A 2005 case report described a young woman who did not respond to twice-daily doses of Rabeprazole. Following further investigations, the cause was revealed to be air swallowing. Therefore, it may be that persistent heartburn can be caused by aerophagia. This is one hypothesis about the relationship between air bubbles and heartburn, as a major symptom of GERD [12].

As was previously noted, in our study, ED was defined as an air column greater than 10 cm in diameter in SV and CV and greater than 15 mm in diameter in V-LES. We observed that if the air bubbles were present in all sections of CT, regardless to their size, the risk for heartburn was greater than when there were only some sporadic air bubbles on CT (OR = 9.42). This finding requires further investigation.

Although there was no significant correlation between regurgitation and the presence of ED, there was a significant correlation between the size of air bubbles in V-LES and the presence of regurgitation. In a review of previous studies, Bredenoord and Weusten [13] showed that the rate of air swallowing was linked to the size of the intragastric air bubble. They also showed that the number of air swallows was linked to the size of the intragastric air.

Further, although there was no significant correlation between regurgitation and the size of bubbles in other parts of the esophagus, the notable differences between the average size of the air column in patients with regurgitation and patients with no regurgitation motivate the continued careful examination of more cases, especially because the average size in patients with regurgitation was higher. Szczesniak showed that regurgitation caused by GERD, and GERD itself can cause air bubbles in the esophagus, thus we can say that regurgitation can also cause air bubbles.

In our research, taking PPIs was considered a factor that confirmed the presence of previous upper gastrointestinal symptoms. Although there was a significant correlation between taking PPIs and the size of the air bubbles, scrutinizing the mean size of the bubbles in patients who used PPIs and those who didn't use the drug revealed some remarkable findings that suggest the need for repeating the study with more cases. In our study, the mean size of bubbles in patients who did not use PPIs was greater than the group who had previously used this drug. This suggests that taking PPIs decreases the size of the air bubbles and that a history of using PPIs could have an influence on some symptoms, which thus may have also had some influence on our results [14]. In a previous study, it was shown that some patients generated a distension-induced contractile response in the upper esophageal sphincter that was related to PPIs [15]. Therefore, it is reasonable to conclude that PPIs can decrease the size and incidence of air bubbles by treating GERD symptoms.

### Limitations

Some minor symptoms of GERD were excluded from analysis, such as toothache that occurred following the consumption of cold or hot food. These minor symptoms were rare and would cause difficulties in a statistical analysis (because of the small amount of data in each group); therefore, we did not include them in our analysis. In a future study, the effects of air bubbles on all symptoms of GERD should be assessed.

## Abbreviations

GERD: Gastroesophageal Reflux Disease; CT: Computed tomography; ED: esophageal dilation; SV: supra ventricle; CV: cardiac ventricle; V-LES: ventricle to lower esophageal sphincter; OR: Odds Ratio; mm: millimeter.

## Competing interests

The authors declare that they have no competing interests.

### Financial competing interests

- In the past five years we have not received reimbursements, fees, funding, or salary from an organization that may have any financial gains from the publication of this manuscript, either in now or in future.

- We do not hold any stocks or shares in an organization that may in any way gain or lose financially from the publication of this manuscript, either now or in the future.

- We do neither hold nor currently applying for any patents relating to the content of the manuscript. We have not received reimbursements, fees, funding, or salary from an organization that holds or has applied for patents relating to the content of the manuscript.

- We have not any financial competing interests.

### Non-financial competing interests

- There are not any non-financial competing interests (political, personal, religious, academic, ideological, intellectual, commercial or any other).

## Authors' contributions

AM: study concept and design; acquisition, read and approved the final manuscript. HR: study concept and design, coordination for the acquisition of data, read and approved the final manuscript. MT: Critical drafting of manuscript and revision of manuscript, read and approved the final manuscript. Sh.Gh: Acquisition, Designing the study, analysis and interpretation of data, writing the manuscript, read and approved the final manuscript.

## Pre-publication history

The pre-publication history for this paper can be accessed here:

http://www.biomedcentral.com/1471-2342/12/2/prepub

## References

[B1] Toghanian S Wahlqvist P Johnson DA Bolge SC Liljas B The burden of disrupting gastro-oesophageal reflux disease: a database study in US and European cohorts Clin Drug Investig 2010 30 3 167 78 10.2165/11531670-000000000-00000 20155989

[B2] Locke GR Talley NJ Fett SL Zinsmeister AR Melton LJ Prevalence and clinical spectrum of gastroesophageal reflux: a population-based study in Olmsted County, Minnesota Gastroenterology 1997 112 5 1448 56 10.1016/S0016-5085(97)70025-8 9136821

[B3] Shaker R Castell DO Schoenfeld PS Spechler SJ Nighttime heartburn is an under-appreciated clinical problem that impacts sleep and daytime function: the results of a Gallup survey conducted on behalf of the American Gastroenterological Association Am J Gastroenterol 2003 98 7 1487 93 10.1111/j.1572-0241.2003.07531.x 12873567

[B4] Vakil N van Zanten SV Kahrilas P Dent J Jones R The Montreal definition and classification of gastroesophageal reflux disease: a global evidence-based consensus Am J Gastroenterol 2006 101 8 1900 20 quiz 43 10.1111/j.1572-0241.2006.00630.x 16928254

[B5] Grainger RGAD Diagnostic radiology: a textbook of medical imaging 2001 UK: Churchill Livingstone

[B6] Goldwin RLHE Proto AV Computed tomography of the mediastinum: normal anatomy and indications for the use of CT Radiology 1977 124 235 41 866645 10.1148/124.1.235

[B7] Bhalla M Silver RM Shepard JA McLoud TC Chest CT in patients with scleroderma: prevalence of asymptomatic esophageal dilatation and mediastinal lymphadenopathy AJR Am J Roentgenol 1993 161 2 269 72 8333359 10.2214/ajr.161.2.8333359

[B8] Ponce J Garrigues V Ramirez JJ Pascual S Arguello L Berenguer J [The clinical significance of the magnitude of esophageal dilatation in idiopathic achalasia] Gastroenterol Hepatol 1996 19 5 235 9 8752563

[B9] Halber MD Daffner RH Thompson WM CT of the esophagus: I. Normal appearance AJR Am J Roentgenol 1979 133 6 1047 50 116493 10.2214/ajr.133.6.1047

[B10] Schraufnagel DE Michel JC Sheppard TJ Saffold PC Kondos GT CT of the normal esophagus to define the normal air column and its extent and distribution AJR Am J Roentgenol 2008 191 3 748 52 10.2214/AJR.07.3455 18716104

[B11] Vakil NB Traxler B Levine D Dysphagia in patients with erosive esophagitis: prevalence, severity, and response to proton pump inhibitor treatment Clin Gastroenterol Hepatol 2004 2 8 665 8 10.1016/S1542-3565(04)00289-7 15290658

[B12] Zentilin P Accornero L Dulbecco P Savarino E Savarino V Air swallowing can be responsible for non-response of heartburn to high-dose proton pump inhibitor Dig Liver Dis 2005 37 6 454 7 10.1016/j.dld.2004.06.020 15893286

[B13] Bredenoord AJ Weusten BL Timmer R Akkermans LM Smout AJ Relationships between air swallowing, intragastric air, belching and gastro-oesophageal reflux Neurogastroenterol Motil 2005 17 3 341 7 10.1111/j.1365-2982.2004.00626.x 15916621

[B14] Dent J Armstrong D Delaney B Moayyedi P Talley NJ Vakil N Symptom evaluation in reflux disease: workshop background, processes, terminology, recommendations, and discussion outputs Gut 2004 53 Suppl 4 iv1 24 15082609 10.1136/gut.2003.034272 PMC1867782

[B15] Szczesniak MM Williams RB Brake HM Maclean JC Cole IE Cook IJ Upregulation of the esophago-UES relaxation response: a possible pathophysiological mechanism in suspected reflux laryngitis Neurogastroenterol Motil 2010 22 4 381 6 e89 10.1111/j.1365-2982.2009.01452.x 20377793

